# Increased coagulation factor XIII activity but not genetic variants of coagulation factors is associated with myocardial infarction in young patients

**DOI:** 10.1007/s11239-019-01856-3

**Published:** 2019-04-10

**Authors:** M. Ambroziak, A. Kuryłowicz, A. Budaj

**Affiliations:** 1 Department of Cardiology, Medical Centre of Postgraduate Education, Grochowski Hospital, Grenadierow 51/59, 04-073 Warsaw, Poland; 2 Department of Human Epigenetics, Mossakowski Medical Research Centre, Polish Academy of Sciences, Pawinskiego 5, 02-106 Warsaw, Poland

**Keywords:** Coagulation factor XIII, Myocardial infarction in young age, Premature coronary artery disease, Thrombospondin-2, Thrombospondin-4

## Abstract

The aim of the study was to investigate the possible role of coagulation factor XIII (FXIII) plasma activity and its gene (*F13A1*) Val34Leu variant as well as thrombospondin-2 gene (*THBS2*) T/G 3′UTR and thrombospondin-4 gene (*THBS4*) Ala387Pro variants in the development of myocardial infarction (MI) in young patients. The studied group consisted of 158 patients aged < 50 years with MI, and the control groups consisted of 150 healthy people aged < 50 years and 202 patients suffering from MI aged ≥ 50 years. Factor XIII activity was measured by photometric assay; genetic variants were determined using the restriction fragment length polymorphism (RFLP) method. FXIII activity was significantly higher in the young MI group compared with young healthy controls and the MI ≥ 50 group (126.2 U/dl vs. 109.6 U/dl, p < 0.0001; 126.2 U/dl vs. 119.8 U/dl, p = 0.01, respectively). FXIII activity did not correlate with *F13A1* gene variants. *F13A1*, *THBS2* and *THBS4* genotypes were equally distributed in all studied groups. There was also no statistically significant differences in the prevalence of the extended CC/TT/GG haplotype of *F13A1/THBS2/THBS4* variants between the young MI group and the young healthy control group and between the young MI group and the MI aged ≥ 50 group. In conclusion, our study revealed that increased FXIII activity is associated with an increased risk of MI in young patients. None of studied single genetic variants—*F13A1* Val34Leu, *THBS2* T/G 3′UTR and *THBS4* Ala387Pro—and the extended CC/TT/GG haplotype of *F13A1/THBS2/THBS4* genes was associated with MI in young age.

## Highlights

- Increased FXIII activity was associated with the incidence of MI in young patients.
- FXIII activity did not correlate with the *F13A1* gene Val34Leu variant.
- Single genetic variants of three coagulation system genes involved in the process of atherosclerosis—Val34Leu of the *F13A1* gene, T/G 3′UTR of the *THBS2* gene and Ala387Pro of the *THBS4* gene—were not associated with MI in young patients.
- The extended CC/TT/GG haplotype of the *F13A1*/*THBS2*/*THBS4* genes, although the most common, was not associated with MI in young people.
- Further studies are required to establish the role of FXIII as a risk factor for MI at a young age.

## Introduction

Atherosclerosis is the result of the interplay between modifiable factors, such as smoking, diet or physical activity, and congenital nonmodifiable factors. Their joined action, which affects the processes of coagulation, fibrinolysis, lipid metabolism and inflammation, is crucial in the pathogenesis of coronary artery disease (CAD), one of the clinical manifestations of atherosclerosis. Variants of genes coding proteins involved in the development of atherosclerosis are thought to be responsible for the occurrence of myocardial infarction (MI), especially in young patients.

The coagulation system plays a pivotal role in the development and progression of atherosclerosis. One of its most important components is coagulation factor XIII (FXIII). This factor plays a crucial role in the equilibrium between thrombus formation and dissolution. FXIII is a protein consisting of two subunits: subunit A, which has an enzymatic transglutaminase function leading to the thrombus formation, and subunit B, which lacks enzymatic activity but is thought to be a carrier of subunit A and thus stabilizes FXIII in the circulation [1]. Subunit A is encoded by the *F13A1* gene at locus 6p25-p24, and subunit B is encoded by the *F13B* gene at locus 1q31-q32.

Increased FXIII levels are associated with the risk of MI in females [2]. On the other hand, FXIII could be involved in tissue healing after MI. FXIII deficiency has been shown to cause cardiac rupture, impair wound healing and aggravate cardiac remodeling in mice with MI [3]. FXIII dynamics could be a prognostic factor of MI evolution and post MI damage responsible for heart failure or death [4].

One of the common genetic variants of the *F13A1* gene is Val34Leu (rs5985: C > A). Data regarding its role in CAD pathogenesis are contradictory. A meta-analysis by Chen et al. suggested that the34Leu variant of the *F13A1* gene could be protective against MI in Caucasians, whereas a meta-analysis by Wang et al. showed that this variant might be associated with MI risk [5, 6]. An increased risk of MI was also demonstrated in Egyptian patients with *F13A1* Val34Leu GT/TT (CA/AA) variants, particularly in association with the fibrinogen β-455 gene G/A polymorphism [7].

Thrombospondin family members are extracellular calcium-binding glycoproteins. They are involved in the regulation of platelet aggregation, inflammatory response, cell proliferation and the control of angiogenesis [8]. Thrombospondin-2 (TSP-2) is encoded by the *THBS2* gene, which includes 23 exons and is located at the 6q27 locus. Thrombospondin-4 (TSP-4), which is encoded by the *THBS4* gene consisting of 29 exons, is located at the 15q14.1 locus. These genes were widely investigated in CAD due to their potential role in atherosclerosis. The results of the Inabe Health and Longevity Study revealed that the rs8089: T > G variant of the *THBS2* gene in the 3′ untranslated region (UTR) was significantly associated with the prevalence of CAD in 170 Japanese subjects [9]. On the other hand, no association was noted between this *THBS2* genetic variant and CAD risk, whereas another thrombospondin family member variant—*THBS4* Ala387Pro—was related to increased CAD risk in the American population [10]. Conversely, the *THBS4* Ala387Pro variant was not associated with CAD and MI in the Chinese Han population [11].

There are no precise data regarding the role of FXIII and different variants of coagulation system genes involved in the process of atherosclerosis in the pathogenesis of premature CAD. The aim of this study was to investigate the possible role of FXIII activity and three genetic variants of the *THBS4* gene, namely, Val34Leu (rs5985: C > A) of *F13A1*, T/G 3′UTR (rs8089: T > G) of *THBS2* and Ala387Pro (rs1866389: G > C), in the development of MI in young patients.

## Materials and methods

### Patients

The examined population included 510 subjects who were participants in a recently published study [12]. The studied group consisted of 158 patients, referred to as cases, admitted to the Department of Cardiology, Medical Centre of Postgraduate Education, Grochowski Hospital (Warsaw, Poland), due to the first episode of acute coronary syndrome (ACS) as defined by clinical symptoms (stenocardial pain); ECG, including non-ST elevation (NSTEMI) and ST elevation myocardial infarction (STEMI); and elevated troponin serum levels. The group consisted of 125 men and 33 women (79.1% and 20.9%, respectively) aged less than 50 (from 26 to 49, mean 43.9 years), including 107 (67.7%) patients with STEMI.

There were two control groups in this study. The first control group, which was the young healthy control group, consisted of 150 people without a history of CAD with a mean age of 42.3 years (30–49). The group included 95 men (63.3%) and 55 women (36.7%) randomly recruited from healthy blood donors in cooperation with the Regional Blood Transfusion Centre (Warsaw, Poland) in 2009–2012. The second control group consisted of 202 patients also hospitalized in the Department of Cardiology, Medical Centre of Postgraduate Education, Grochowski Hospital (Warsaw, Poland), in 2005–2012 due to the first episode of ACS, including 130 (64.3%) patients with STEMI. Patients in this group were older than the studied group, i.e., ≥ 50 years (from 50 to 92, mean 65.1 years). This group included 130 men (64.3%) and 72 women (35.7%).

All participants of the study provided written informed consent. The Ethical Committee of the Medical Centre of Postgraduate Education approved the study protocol. The investigation conforms to the principles outlined in the Declaration of Helsinki.

### FXIII activity and biochemical parameter measurements

Blood samples were taken after a 12-h overnight fast on the day of discharge from the hospital (5th–10th day from the incidence of ACS) for MI patients. The test was performed in platelet-poor plasma (PPP) obtained from citrate-treated blood by two immediate sequential centrifugations performed at room temperature at 1500 ×*g* for 15 min. The PPP was stored at –70 °C until use.

FXIII activity was determined by photometric assay using the Berichrom^®^ FXIII test kit and Standard Human Plasma as a calibrator (Siemens Healthcare Diagnostics Products GmbH, Marburg, Germany) on a BCS XP^®^ analyzer (Siemens Healthcare Diagnostics Products GmbH, Marburg, Germany) in accordance with the manufacturer’s protocol. Activator reagent contained bovine thrombin, clot inhibitor, calcium chloride, hexadimethrine bromide, bovine albumin, BICINE buffer, and NADH reagent (NADH 0.5 g/l). Detection reagent contained glutamate dehydrogenase (GLDH 20 IU/ml), the synthetic peptide as FXIII substrate (2.4 g/l), ADP, glycine ethyl ester, α-ketoglutarate, bovine albumin, and HEPES buffer. The assay is based on measuring the ammonia released during the transglutaminase reaction by the nicotinamide adenine dinucleotide phosphate (NAD[P])H-dependent glutamate dehydrogenase reaction spectrophotometrically at 340 nm [13]. The results were expressed as a percentage of the norm. The reference interval was 70–140% of the norm (values 70–140 U/dl or 0.70–1.4 U/ml).

Biochemical analyses, including plasma glucose, total cholesterol, HDL and LDL cholesterol and triglyceride (TG) concentrations, were performed in fasting blood samples by standard enzymatic methods using COBAS INTEGRA 800 regents and equipment (Roche Diagnostics Gmbh).

### Genotyping

Genomic DNA was extracted from peripheral blood mononuclear cells using the salting-out method [14]. Genotyping of the selected variants was performed by polymerase chain reaction (PCR) amplification, followed by digestion with a proper restriction enzyme (restriction fragment length polymorphism method—RFLP).

**Fig. 1:**
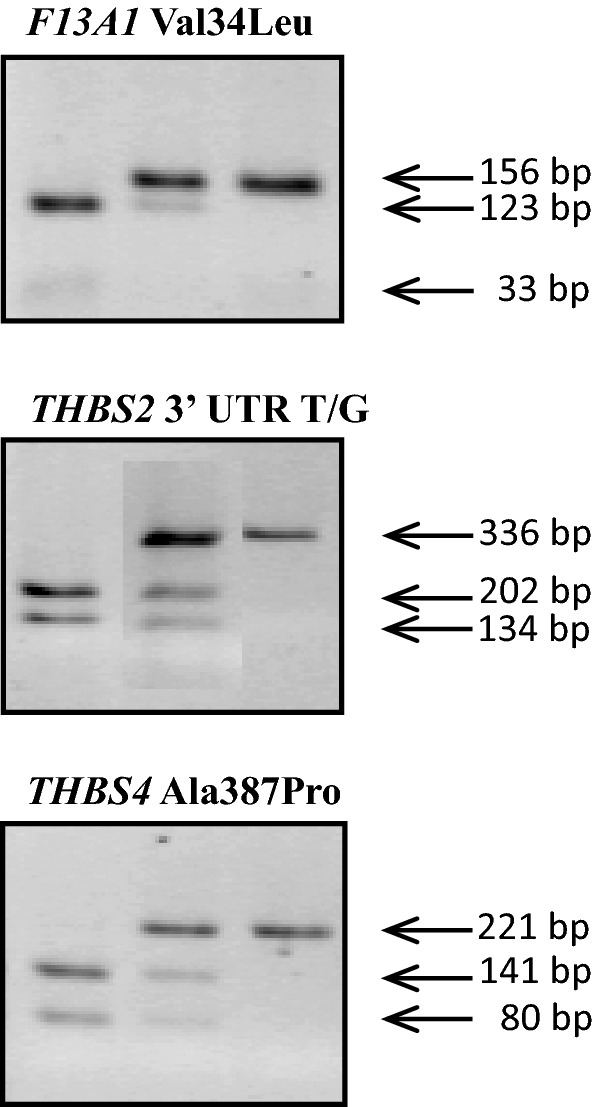
Restriction fragments of the studied gene variants as assessed by agarose gel electrophoresis. *Bp* base pairs, *F13A1* coagulation factor XIII gene, *UTR* untranslated region, *THBS2* thrombospondin 2 gene, *THBS4* thrombospondin 4 gene

PCR conditions were as follows: initial denaturation 94 °C for 5 min; 35 cycles of 94 °C for 30 s, annealing at temperatures of 57 °C (*F13A1*), 65 °C (*THBS2*) or 60 °C (*THBS4*) for 30 s, and extension at 72 °C for 30 s; and a final step at 72 °C for 5 min. Each 12.5 µl reaction contained 50 ng of DNA, 2.0 mM MgCl_2_ (for *F13A1* and *THBS2*) or 1.5 mM MgCl_2_ (for *THBS4*), 10 pmol of each primer, 0.25 mM of each deoxynucleoside triphosphate and 1 unit of *Taq* polymerase (Invitrogen Carlsbad, USA) in a corresponding buffer. Then, 2.5 µl of the PCR product was digested with the restriction enzymes MseI, DdeI and AvaII for *F13A1*, *THBS2* and *THBS4*, respectively. The restriction site for MseI for the *F13A1* Val34Leu variant was created with the use of the mutated reverse primer (NCBI Reference Sequence: NT_007592.16, underlined in Table 1). The obtained restriction fragments were visualized on a 3% agarose gel (Fig. 1). Genotyping of the *F13A1* variant was performed in 143 of 158 patients with MI < 50 due to the lack of DNA material.

**Table 1:** Primers, PCR conditions, restriction enzymes and fragments of the studied variants

Gene	Variant	Accession no	Primers	MgCl_2_ [mM]	PCR product	Restriction enzyme	Alleles
*F13A1*	Val34Leu C/A	rs5985	F: 5′GACCTTGTAAAGTCAAAAATGTC 3′	2.0	156 bp	*Mse*I	C: 156 bp
			R: 5′GCTCATACCTTGCAGGTTGACGCCCCGGGGCATTA 3′				A: 123 bp; 33 bp
*THBS2*	3′UTR T/G	rs8089	F: 5′CTGTGCATGCCATGGTCCCTAGA 3′	2.0	363 bp	*Dde*I	G: 336 bp; 27 bp
			R: 5′TATCATAATGGCTTATGCACAGTATTCCCTTCA 3′				T: 202 bp; 134 bp; 27 bp
*THBS4*	Ala387Pro C/G	rs1866389	F: 5′AATTCCGCATCTTCACTTCAC 3′	1.5	221 bp	*Ava*II	G: 221 bp
			R: 5′ AACCGGTTCTGCTTTGATAAC 3′				C: 141 bp; 80 bp

The restriction site for MseI in the *F13A1* Val34Leu variant was created using the mutated reverse primer (NCBI reference sequence: NT_007592.16)

*Bp* base pairs, *F13A1* coagulation factor XIII gene, *UTR* untranslated region, *THBS2* thrombospondin 2 gene, *THBS4* thrombospondin 4 gene

### Statistical analysis

The comparison of the studied groups, including FXIII activity and all clinical data, was performed with the Mann–Whitney U test using the Statistica software package (StatSoft Inc., Tulsa, USA) and Student’s *t* test for age. P-value significance was established at 0.05.

The distribution of genotypes was analyzed in three models of inheritance: dominant, recessive and co-dominant. Under each model, the odds ratio (OR) with a 95% confidence interval (CI) and the P-value for an association were calculated. Calculations were performed using the Web-Assotest program (available at http://www.ekstroem.com/assotest/assotest.html), and power analysis was performed using the DSS software available online (http://www.dssresearch.com). Hardy–Weinberg equilibrium was assessed using the χ^2^ test and was calculated based on the entire cohort. Χ^2^ for each studied group (MI < 50, MI ≥ 50, young healthy controls) was determined separately.

Moreover, the multivariate logistic regression model was applied to investigate the association between independent covariates and binary endpoints. The following predictors were included in the model: gender, smoking, hypertension, DM2, total cholesterol, LDL cholesterol, HDL cholesterol, triglyceride levels, BMI, creatinine, glucose at admission, fasting glucose, the presence of depression, education level, type of work and civil status. Individual genetic variants and gender interactions were included in the model to assess possible differences between males and females in terms of the effects on endpoint variants. A stepwise selection procedure was applied with a 10% level for staying within the model. All independent covariates except the genetic variant were considered in the selection procedure. The tests were two-sided, and a 5% level of significance was used. Association between significant predictors and the binary endpoint was expressed using the odds ratio and 95% confidence interval.

## Results

### FXIII activity and *F131A* gene Val34Leu variants

**Fig. 2:**
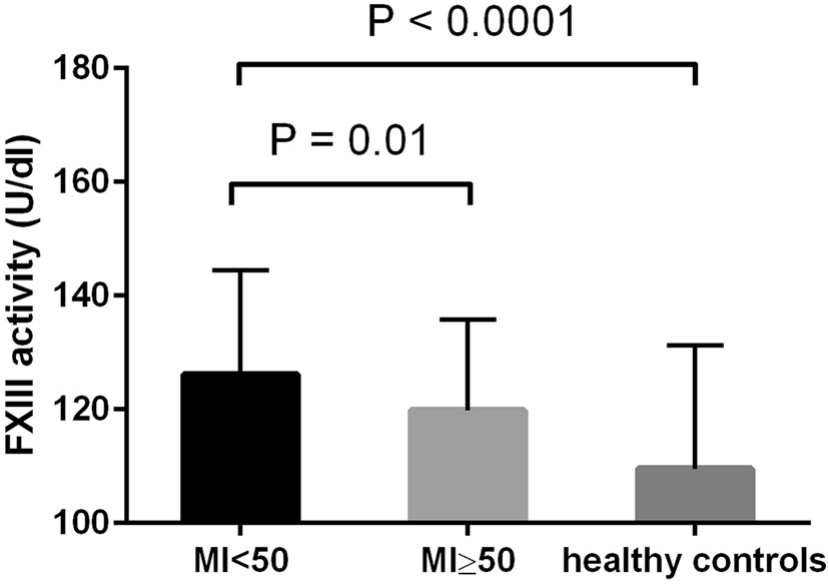
Median FXIII activity in the young MI < 50 group (126.2 U/dl, 25–75 quartile 107.1–144.4), MI ≥ 50 group (119.8 U/dl, 25–75 quartile 98.5–135.8), and young healthy control group (109.6 U/dl, 25–75 quartile 96.7–131.3). The differences between the MI < 50 and MI ≥ 50 groups as well as the young healthy control group were statistically significant (p = 0.01 and p < 0.0001, respectively). FXIII—coagulation factor XIII, MI < 50—patients with myocardial infarction aged < 50 years, MI ≥ 50—patients with myocardial infarction aged ≥ 50 years

Median FXIII activity was significantly higher in the young MI group compared with the young healthy group (126.2 U/dl vs. 109.6 U/dl, p < 0.0001) and MI ≥ 50 group (126.2 U/dl vs. 119.8 U/dl, p = 0.01) (Fig. 2). The percentage of patients with increased (> 140 U/dl) FXIII activity was higher in the young MI group (30.8%) compared with the young healthy group (14.7%) and MI ≥ 50 group (18.8%).

There were no differences in median FXIII activity for all studied patients between AA and CC carriers (114 vs. 117.53 U/dl, p = 0.904), AA and CA carriers (1146 U/dl vs. 121.8 U/dl, p = 0.267), CA and CC carriers (121.8 U/dl vs. 117.5 U/dl, p = 0.155), as well as AA + CA and CC carriers (120.25 U/dl vs. 117.5 U/dl, p = 0.265) and CC + CA and AA carriers (119.7 U/dl vs. 114.6 U/dl, p = 0.568). There were also no differences in FXIII activity between particular *F13A1* genotype carriers analyzed separately in the MI < 50, MI ≥ 50 and young healthy groups. The percentage of patients with increased (> 140 U/dl) FXIII activity in the particular genotype groups were as follows: 16.7% in AA, 23.3% in CA, 20.4% in CC carriers, 21.6% in CC + CA and 21.7% in AA + CA carriers, without any statistically significant differences between these groups.

### Distribution of particular genotypes in the studied groups

Genotypes of the *F13A1*, *THBS2* and *THBS4* genes were equally distributed between the MI < 50 group and the MI ≥ 50 group as well as between the MI < 50 group and the young healthy control group (Table 2). A multivariate regression analysis showed that none of the studied genotypes could be an independent risk factor for CAD in young patients.

**Table 2:** Distribution of the studied variants of the *F13A1*, *THBS2* and *THBS4* genes

Gene	Variant	Genotype	MI < 50 n (%)	MI ≥ 50 n (%)	Healthy controls n (%)
*F13A1*	Val34Leu	CC	76 (53.1)	94 (46.5)	85 (56.7)
		CA	48 (33.6)	79 (39.1)	53 (35.3)
		AA	19 (13.3)	29 (14.4)	12 (8.0)
*THBS2*	T/G	TT	91 (57.6)	127 (62.9)	90 (60.0)
		TG	59 (37.3)	64 (31.7)	50 (33.3)
		GG	8 (5.1)	11 (5.4)	10 (6.7)
*THBS4*	Ala387Pro	GG	106 (67.1)	133 (65.8)	107 (71.3)
		GC	47 (29.7)	58 (28.7)	38 (25.3)
		CC	5 (3.2)	11 (5.4)	5 (3.3)

The prevalence of particular genotypes in three groups of patients did not differ

*F13A1* coagulation factor XIII gene, *MI < 50* patients with myocardial infarction aged < 50 years, *MI ≥ 50* patients with myocardial infarction aged ≥ 50 years, *THBS2* thrombospondin 2 gene, *THBS4* thrombospondin 4 gene

Based on the prevalence of particular genotypes, we performed further analysis of the extended haplotypes of the *F13A1/THBS2/THBS4* genes variants The most common extended haplotype was CC/TT/GG. There was no statistically significant difference in the prevalence of this haplotype between the MI < 50 group and the MI ≥ 50 group (18.9% vs. 18.7%; OR 1.0, 95% CI 0.59–1.75, p = 1.0) as well as between the MI < 50 group and the young healthy control group (18.9% vs. 26.8%; OR 0.63, 95%CI 0.37–1.0, p = 0.106) (Table 3).

**Table 3:** Distribution of homozygotic haplotypes of the three studied gene variants Val34Leu *F13A1*, T > G *THBS2* and Ala387Pro *THBS4* in the studied group of young MI < 50 years compared with MI ≥ 50 years and the healthy control group

*F13A1/THBS2/THBS4*	MI < 50 n (%)	MI ≥ 50 n (%)	Young healthy controls n (%)
Haplotypes	a	b	c
CC/TT/GG	27 (18.9)	38 (18.7)	41 (26.8)
AA/TT/GG	9 (6.3)	12 (5.9)	6 (3.9)
CC/GG/GG	4 (2.8)	2 (0.9)	3 (1.9)
CC/TT/CC	2 (1.4)	3 (1.5)	1 (0.6)
AA/TT/CC	1 (0.7)	1 (0.5)	0
AA/GG/GG	0	0	0
AA/GG/CC	0	0	0
CC/GG/CC	0	0	0

The differences between groups were not statistically significant

*CI* confidence interval, *FXIII* coagulation factor XIII, *F13A1* coagulation factor XIII gene, *MI < 50* patients with myocardial infarction aged < 50 years, *MI ≥ 50* patients with myocardial infarction aged ≥ 50 years, *ns* not significant, *OR* odds ratio, *THBS2* thrombospondin 2 gene, *THBS4* thrombospondin 4 gene

There were no differences in the median values of FXIII activity between carriers of the extended CC/TT/GG haplotype and carriers of other haplotypes (116.4 U/dl (25–75 quartile 100.1–136.7) and 120 U/dl (25–75 quartile 99.3–136.8), respectively, p = 0.622) (Table 4).

**Table 4:** Distribution of CAD risk factors and FXIII activity in carriers of the homozygotic CC/TT/GG haplotype of the *F13A1/THBS2/THBS4* genes and patients with other haplotypes

	CC/TT/GG haplotype n = 116	Other haplotypes n = 394	p
BMI median (25–75 quartile)	26.6 (24.2–29.4)	27.7 (24.7–30.5)	0.083
Family history of MI/stroke; n (%)	20 (17.2)	61 (15.5)	0.438
Smoking n (%)	82 (70.7)	260 (66)	0.446
Hypertension n (%)	51 (44)	207 (52.5)	0.505
Diabetes mellitus n (%)	21 (18.1)	94 (23.8)	0.716
Depression n (%)	11 (9.5)	22 (5.6)	0.509
Total cholesterol mg/dl median (25–75 quartile)	198 (171.5–237)	202 (174–228)	0.567
HDL mg/dl median (25–75 quartile)	42 (38–51)	37 (36–51)	0.342
LDL mg/dl median (25–75 quartile)	128 (98–160)	131 (102–153)	0.778
TG mg/dl median (25–75 quartile)	127 (81.5–178.5)	124 (90–180)	0.576
Glucose at admission mg/dl median (25–75 quartile)	131.5 (109–168)	131 (110–165)	0.986
Fasting glucose mg/dl median (25–75 quartile)	98 (90–118)	100 (90–118)	0.735
FXIII U/dl median (25–75 quartile)	116.4 (100.1–136.7)	120.0 (99.3–136.8)	0.622

No statistically significant differences between groups were revealed

*BMI* body mass index, *FXIII* coagulation factor XIII, *F13A1* coagulation factor XIII gene, *GFR* glomerular filtration rate, *HDL* high-density lipoprotein, *LDL* low-density lipoprotein, *MI* myocardial infarction, *TG* triglycerides, *THBS2* thrombospondin 2 gene, *THBS4* thrombospondin 4 gene

### Association between the *F13A1, THBS2* and *THBS4* genetic variants and classical CAD risk factors

There were no differences in BMI, family history of premature atherosclerosis at a young age (MI or stroke in men aged < 55 or in women aged < 65 years of age), smoking, arterial hypertension, diabetes mellitus, depression, glucose, triglycerides, and median plasma concentrations of total cholesterol and LDL and HDL cholesterol among the AA, AC and CC genotypes of the Val34Leu variant of the *F13A1* gene. Similarly, there were no differences in the distribution of the above-mentioned classical CAD risk factors among the TT, TG and GG genotypes of the T/G 3′UTR of the *THBS2* gene.

Regarding Ala387Pro variants of the *THBS4* gene, there was a higher prevalence of hypertension for GG + GC compared to CC carriers (95.8% vs. 4.2%, p = 0.016) and a lower prevalence of depression for CC + GC carriers compared with to GG carriers (31.9% vs. 68.1%, p = 0.019). There were no other differences between particular genotypes of the *THBS4* gene Ala387Pro variants.

Analyzing the extended CC/TT/GG haplotype of *F13A1*/*THBS2*/*THBS4* and other haplotypes, there were no differences regarding classical risk factors, including BMI, family history of premature atherosclerosis at a young age, smoking, arterial hypertension, diabetes mellitus, depression, glucose, triglycerides, and median plasma concentration of total, LDL and HDL cholesterol (Table 4).

## Discussion

In our study, there was statistically higher plasma activity of FXIII in young MI patients aged < 50 years compared with young healthy people at the same age and patients with MI aged ≥ 50 years. This is the second study regarding FXIII activity in MI in young patients. A recently published study revealed significantly elevated FXIII activity in very young patients below 40 years of age, but the increase was limited to patients with STEMI [15]. Our study included patients with STEMI and NSTEMI.

Several studies mentioned above suggest the role of FXIII in the pathogenesis of CAD [2–4]. Moreover, higher FXIII activity was associated with a higher severity of CAD in a group of 191 UK Asians [16]. A positive correlation of FXIII activity with the severity of aortic valve stenosis was also reported [17].

The data regarding the role of the Leu34Val variant of the *F13A1* gene in the development of premature atherosclerosis remain controversial. The frequency of single nucleotide point mutations leading to the presence of Leu in the amino acid chain was significantly lower in the group of 130 patients with MI aged < 60 compared with the healthy control group of individuals of the same age [18]. Similarly, the Greek study indicated that the *F13A1* Val34Leu variant (T allele) had a protective effect against the development of premature MI in a very young population aged < 36 years [19].

On the other hand, the *F13A1* Val34Leu GT (CA) genotype and T (A) allele were shown to have a protective effect in the unstable angina group of very young patients aged < 40 years but not in MI patients [20]. Nevertheless, the *F13A1* gene T variant was also associated with arterial ischemic stroke in children, particularly in combination with several coagulation factor gene variants [21]. Although the modified fibrin phenotype seems to be related to premature CAD, there was no association between the *F13A1* Val34Leu genetic variant and MI in young patients in other studies [22, 23]. Nevertheless, the most recently published meta-analysis revealed that *F13A1* Val34Leu variant was associated with CAD risk, especially MI, without differences in age [24].

There were no differences in the prevalence of *F131A* genotypes among the MI < 50, MI ≥ 50 and young healthy groups in our study. Moreover, FXIII activity did not correlate with the *F13A1* gene Val34Leu variant. These data are in opposition with several studies regarding FXIII activity and the *F13A1* gene Val34Leu variant. Kohler et al. indicated a stepwise increase in FXIII activity when Leu was present in healthy Caucasian subjects with a mean age of 56.1 years [25]. FXIII activity is also higher in Leu34 carriers compared with Val34Val genotype carriers in 80 cases derived from a cohort of MI patients with a mean age of 66.5 years [26].

The differences between these studies and ours could be the effect of distinct characteristics of the study populations with respect to health status (presence of MI) and age, which seem to be crucial. The close proximity of the mutation in the thrombin activation site is thought to be responsible for changes in FXIII activity [27]. Such interplay may be affected by genomic as well as nongenomic and epigenetic regulation and modified by age, previous treatment or comorbidities.

Moreover, the involvement of FXIII in the development of atherosclerosis does not seem to be associated with its action as a coagulation factor but rather as an inflammation modulator. FXIII is a key regulator of fibrinolysis and is present in platelets, monocytes and macrophages. FXIII binds platelets via GPIIb/IIIa and mediates the endothelial cell–platelet interaction [28]. FXIII induces monocyte migration and proliferation [29]. FXIII was also shown to regulate gene expression in alternatively activated monocyte-derived macrophages [30].

Similar to the *F13A1* gene Val34Leu variant, there are contradictory data regarding the role of the *THBS2* and *THBS4* gene variants in premature CAD pathogenesis. The missense variant Ala387Pro of *THBS4* showed a strong association with MI in young (men aged < 45, women aged < 50 years) individuals carrying the Pro allele, whereas a variant in the 3′ untranslated region of the *THBS2* gene with a change of thymidine to guanine seemed to have a protective effect against MI in homozygous individuals [31]. Homozygosity for the *THBS2* G/T variant and the *THBS4* Ala387Pro variant was significantly associated with the reduced risk of premature MI [32].

In our study, genotypes of the *F13A1*, *THBS2* and *THBS4* gene variants were equally distributed in all studied groups. This finding does not indicate that these genotypes could be independent risk factors for CAD development in young people. Moreover, regarding our results, the most common extended haplotype (CC/TT/GG) of the *FXIII/THBS2/THBS4* gene variants was also not associated with the risk of MI in young patients. These findings are consistent with data indicating that the influence of genetic variants on CAD development is not the result of a single gene action but the effect of several gene-to-gene and gene-to-environmental risk factors interplay with differing interactions among populations, races and genders.

The major limitation of our study is the relatively small number of patients. On the other hand, the high homogeneity of the groups, which were limited to Polish populations of a Caucasian race, could be of value regarding the above-mentioned data, indicating significant population and racial differences in the involvement of genetic variants in the pathogenesis of CAD.

In conclusion, our study revealed for the first time that increased FXIII activity was associated with the incidence of MI in young patients. Genetic variants of three coagulation system genes involved in the process of atherosclerosis—Val34Leu of the *F13A1* gene, T/G 3′UTR of the *THBS2* gene and Ala387Pro of the *THBS4* gene—were not associated with MI in young patients. The extended CC/TT/GG haplotype of the *F13A1*/*THBS2*/*THBS4* genes, although the most common, was not associated with MI in young people.

## Abbreviations

CAD: Coronary artery disease
FXIII: Coagulation factor XIIIA
*F13A1*: Coagulation factor XIII gene
GFR: Glomerular filtration rate
HDL: High-density lipoprotein
LDL: Low-density lipoprotein
MI: Myocardial infarction
PCR: Polymerase chain reaction
RFLP: Restriction fragment length polymorphism
TG: Triglycerides
*THBS2*: Thrombospondin-2 gene
*THBS4*: Thrombospondin-4 gene
TSP-2: Thrombospondin-2
TSP-4: Thrombospondin-4
UA: Unstable angina
UTR: Untranslated region
